# Bowel Damage at Diagnosis Using the Lémann Index Score in Saudi Arabian Patients With Crohn's Disease

**DOI:** 10.7759/cureus.10912

**Published:** 2020-10-12

**Authors:** Hajar Halawani, Ahmed Abduljabbar, Mohammad Wazzan, Dalia Abdulmonem Hashem, Cedric Baumann, Amandine LUC, Laurent Peyrin-Biroulet, Omar I Saadah, Mahmoud Mosli

**Affiliations:** 1 Radiology, King Abdulaziz University, Jeddah, SAU; 2 Gastroenterology, University Hospital of Nancy, France, FRA; 3 Gastroenterology, Nancy University Hospital, France, FRA; 4 Pediatric Gastroenterology, King Abdulaziz University Hospital, Jeddah, SAU; 5 Gastroenterology, King Abdulaziz University Hospital, Jeddah, SAU

**Keywords:** crohn’s disease, lémann index, enterography, endoscopy, resection, saudi arabia

## Abstract

Background

Advanced bowel damage caused by Crohn’s disease (CD) in the form of strictures and penetrating lesions has been associated with future surgical resection. However, in general, the degree of bowel damage in patients with CD is not examined at the time of diagnosis, and the natural history of CD may differ phenotypically between patients from Arabic countries as compared to patients from Europe and North America. Thus, we aimed to assess the degree of structural bowel damage in Saudi Arabian CD patients at diagnosis. We used the Lémann Index (LI) score, an instrument that measures cumulative digestive tissue damage by magnetic resonance enterography (MRE) and endoscopy, to establish any possible association between the duration of symptoms and the degree of bowel damage.

Method

This retrospective study was conducted by reviewing the data of all CD patients following up at King Abdulaziz University Hospital (KAUH) that were investigated by endoscopy and MRE at baseline. MRE-LI was calculated by scoring previous surgery, disease location and extension, and intestinal complications. A LI score of >2.0 was set as the cut-off point for bowel damage. Descriptive statistics were used to provide an overview of demographic and clinical characteristics, and hypothesis testing was applied to identify associations.

Result

Eighty-three patients with CD were included in this study. Fifty point six percent (50.6%) of the cohort comprised females and the median age was 27 years. With regards to CD location and extension, 34.9% showed ileal disease (L1), 9.6% showed colonic CD (L2), whereas 55.4% had ileocolonic involvement (L3). Moreover, 48.2% of patients presented with non-complicated behavior (B1), 25.3% had at least one stricture (B2), and 26.5% showed a penetrating phenotype (B3). Perianal CD was observed in 2.4% of subjects and 62.7% had undergone bowel resection. Mean LI was 2.4 (±2.6) with 34 patients (39.8%) exhibiting an LI score indicative of advanced bowel damage at the time of diagnosis. The duration of symptoms did not correlate with the degree of bowel damage according to the LI score.

Conclusion

A significant proportion of patients with CD presented with advanced bowel damage at the time of diagnosis, suggesting that a severe form of CD may be endemic in Saudi Arabia.

## Introduction

Crohn's disease (CD) is a chronic inflammatory disease with progressive involvement of the gastrointestinal tract (GI) leading to bowel damage. Multiple complications and extra-intestinal manifestations (EIMs) may occur during the clinical course of the disease. Advanced bowel damage in the form of strictures or penetrating lesions are associated with future surgical bowel resection [1-2]. The clinical onset and natural course of CD is highly unpredictable owing to fluctuation in the degree of bowel damage that patients initially demonstrate [1]. Up to 60% of patients with CD will require surgical intervention, sometimes during the first year of the disease [3]. Therefore, predicting the risk of surgery and monitoring the degree of bowel damage is crucial in the management of CD to guide an effective personalized therapeutic decision.

The Lémann Index (LI) has been proposed to be an important tool to determine bowel damage [4]. It measures cumulative digestive tissue damage in patients with CD based on the severity and extent of the disease by assessing the presence of strictures, penetrating lesions, and history of surgical resection according to measurements calculated by magnetic resonance imaging (MRI) of the small bowel and gastrointestinal endoscopy; this can be used to predict long-term disability. The LI allows clinicians to monitor disease progression and optimize the impact of different therapeutic strategies in patients with CD [4].

Current literature in gastroenterology has revealed an increase in the incidence of CD among Arabic populations, such as from 3.1/100,000 person-years in 2003 to 10.6/100,000 person-years in 2008 [5]. Another study has shown an increasing trend in the incidence of inflammatory bowel disease (IBD), including CD, over time in Saudi Arabian children with IBD [6]. Broadly, however, there are limited data on the natural history of CD and degree of bowel damage at the time of diagnosis in Arabic populations.

Delineating the disease course and severity of CD among Arabic populations at the time of diagnosis is crucial for developing treatment plans that are specific to this group. Therefore, we assessed the degree of structural bowel damage at the time of diagnosis using the LI score and examined the possible association between the duration of symptoms and the degree of bowel damage in Saudi patients diagnosed with CD.

## Materials and methods

We conducted a retrospective review study, following approval of the Research Committee at the Biomedical Ethics Unit at King Abdulaziz University (KAU), Jeddah, Saudi Arabia. Participants of the study comprised all patients diagnosed with CD between 2010 and 2018 who were investigated by endoscopy and magnetic resonance enterography (MRE) at the time of diagnosis. The King Abdulaziz University Hospital (KAUH) inflammatory bowel disease information system (IBDIS) registry was searched for patients with CD that fulfilled the study inclusion criteria. The diagnosis of CD was based on radiographic, endoscopic, and histologic criteria [7].

Study variables

Data were collected using a standardized questionnaire at the time of diagnosis. The following data were extracted for each patient: age, sex, previous history of intestinal surgery, and prior history of abdominal collections (phlegmon or abscess) or bowel obstruction. The phenotype of CD was assessed by the Montreal classification of inflammatory bowel disease, according to the location (L1 to L4) and behavior of disease (B1 to B3) at the time of diagnosis [8]. Laboratory results of biomarkers were collected, including C-reactive protein (CRP), erythrocyte sedimentation rate (ESR), hemoglobin (Hb), platelets, leukocytes, and albumin. Morphologic evaluation of CD lesions by endoscopy and abdominal cross-sectional imaging was ascertained. All medications used during the follow-up were also recorded, including corticosteroids, 5-aminosalicylic acid (5ASA) derivatives, immunomodulators, and anti-tumor necrosis factor (anti-TNF) agents.

Radiological assessment

The imaging test performed at the time of diagnosis was selected as the index-imaging test. Two gastroenterologists and two specialized radiologists generated the LI score.

The methods we used for the calculation of the LI at diagnosis in order to predict the risk of early surgery in patients with CD was based on the severity scale for damage lesions (stricturing lesions, penetrating lesions) for each investigational method, the coefﬁcients for structuring, and penetrating lesions of each grade of severity and the model to calculate the LI [4].

Lémann Score = 2 * Sum ‘Upper tract’ + 5 * Sum ‘Small Bowel’ + 3.5 * Sum ‘Colon and rectum’ + 3.5 * Sum ‘Anus’

An LI score of > 2.0 was used as a cut-off point for advanced bowel damage.

Study outcomes

The primary study outcome was to determine the proportion of CD patients with advanced bowel damage according to the LI score at the time of diagnosis. The main secondary study outcome was to establish any possible correlation between bowel damage and the duration of symptoms.

Statistical analysis

The descriptive statistics of the median and interquartile range (IQR) were used for continuous variables and frequencies, and percentages for categorical variables. Categorical variables were tested by the chi-square and Fisher’s exact tests; two by two comparisons were planned where necessary. Continuous values were compared using the Mann-U Whitney or Kruskal-Wallis test where appropriate. Hypothesis testing was employed to examine the association between duration of symptoms and degree of bowel damage according to the LI score and to identify predictors of advanced bowel damage; a p-value of < 0.05 was set as statistically significant. The SAS v9.5 software (SAS Institute, NC Cary) was used in the statistical analysis.

## Results

Baseline characteristics

A total of 643 patients with confirmed IBD were screened in the IBDIS registry, of which 348 were found to have CD. After the application of the study’s inclusion criteria, 83 patients with CD were incorporated. Females comprised 50.6% of the cohort and the median age was 27 years (IQR, 19-35). With regards to CD location and extension, 34.9% showed ileal disease (L1), 9.6% showed colonic CD (L2), whereas 55.4% had an ileocolonic disease (L3). Moreover, 48.2% of the patients presented a non-complicated behavior (B1), 25.3% had at least one stricture (B2), and 26.5% showed a penetrating phenotype (B3). Perianal CD was observed in 2.4% of the total cohort. Fifty-two patients (62.7%) had undergone previous bowel resections, 44 patients (53.0%) had a previous history of perforations, 42 patients (50.6%) had a previous history of abscess collections, and 38 patients (45.8%) had a previous history of intestinal obstructions. Biomarkers revealed the elevation of albumin (mean value of 27.7 g/dL), leukocytes (mean value of 8.9 K/uL), and inflammatory markers, including CRP (mean value of 33.9 mg/L) and ESR (mean value of 26.7 mm/H). With regards to endoscopic findings at the time of diagnosis, ulcerations within the terminal ileum (TI) were found in 43 patients (82.7%). Stenosis of the TI was found in 21 patients (40.4%) and fistula openings at the TI were found in 31 patients (20.6%). Regarding treatment, 42.2% of patients were treated with mesalamine, 74.7% with thiopurines, 3.8% with methotrexate (MTX), 61.5% with anti-TNF-alpha therapy, and 71.2% with corticosteroids. The main baseline features of the study population are listed in Table 1.

**Table 1:** Baseline characteristics of patients with Crohn’s disease (n=83) WBC: white blood cell; CRP: C-reactive protein; ESR: erythrocyte sedimentation rate

	Number (%) or means ± SD
Demographics	
Age at diagnosis (years)	28.5±12
Female gender	42 (50.6%)
Montreal Classification	
Age category A1: <17 A2: 17-40 A3: >40	26 (31.3%) 52 (62.7%) 5 (6%)
Disease location L1: Terminal Ileal L2: Colonic L3: Ileo-colonic	29 (34.9%) 8 (9.6%) 46 (55.4%)
Disease behavior B1: Non-stricturing, non-penetrating B2: Stricturing B3: Penetrating	40 (48.2%) 21 (25.3%) 22 (26.5%)
Endoscopic findings	
Deep ulceration at the terminal ileum Stenosis and stricture at terminal ileum Fistula opening at the terminal ileum	43 (82.7%) 21 (40.4%) 13 (20.6%)
Laboratory findings	
WBCs (K/uL)	8.9±3.3
Hemoglobin (g/dL)	11.5±2.4
Platelets (K/uL)	407±180.5
Albumin (g/dL)	27.7±8.6
CRP (mg/L)	33.9±35.5
ESR (mm/H)	26.7±19.2
Medications	
Budesonide	12 (14.6%)
Prednisolone	47 (56.6%)
5-Amino salicylic acid (5ASA)	35 (42.2%)
Azathioprine (AZA)	62 (74.7%)
Methotrexate (MTX)	3 (3.8%)
Adalimumab	36 (43.4%)
Infliximab	15 (18.1%)
Complications	
Perforation	44 (53%)
Intestinal obstruction	38 (45.8%)
Abscess	42 (50.6%)
Bowel resection	52 (62.7%)

Study outcomes

Bowel Damage at Diagnosis

The mean LI score was 2.4 (SD=2.6; range 0-15.5). Thirty-four patients (39.8%) exhibited an LI score indicative of advanced bowel damage at diagnosis. Components of the LI distributed by organ and bowel segment in relation to the type of lesion are outlined in Table 2.

**Table 2:** Distributions of damage components including number of segments with stricturing or penetrating lesions of the most severe grade

	Stricturing lesions					Penetrating lesions				
Organ	No. of segments	N	Maximal grade			No. of segments	N	Maximal grade		
			1	2	3			1	2	3
Upper tract	0	83				0	83			
	1		0	0	0	1		0	0	0
	2		0	0	0	2		0	0	0
	3		0	0	0	3		0	0	0
Small bowel	0	12				0	55			
	1	42	2	32	8	1	16	0	3	13
	2	18	1	8	9	2	9	0	1	8
	3	8	0	6	2	3	3	1	0	2
	>3	3	0	2	0	>3	-			
Colon/Rectum	0	58				0	79			
	1	17	3	11	3	1	3	0	1	2
	2	6	0	6	0	2	1	0	1	0
	3	2	0	2	0	3	-			
	>3	-				>3	-			
Anus	0	81				0	82			
	1	2	0	2	0	1	1	0	1	0

Duration of Symptoms and Bowel Damage

**Figure 1:**
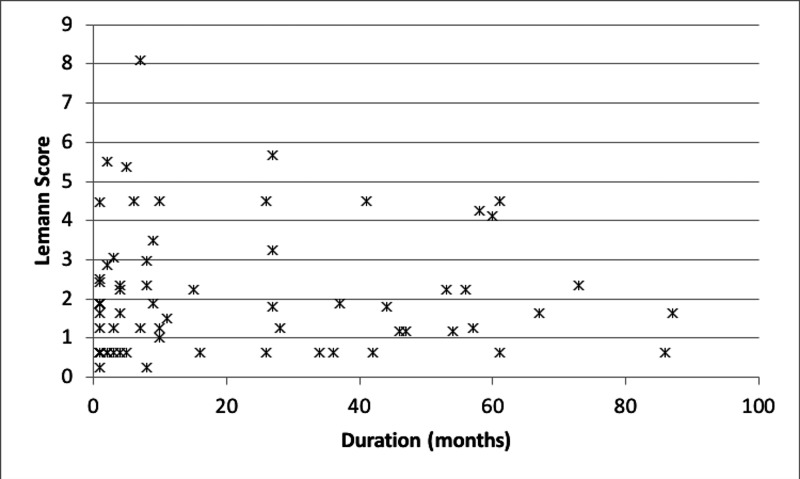
Correlation between degree of bowel damage and duration of symptoms

We evaluated the association between LI scores and the duration of CD symptoms in months. No significant difference in the proportion of patients with advanced bowel damage was noted between patients with a duration of symptoms of ≤18 months and patients with symptoms that were >18 months (34% vs, 38.4%, P=0.79) (Table 3; Figure 1).

**Table 3:** Comparisons of the Lémann score according to the duration of symptoms * Standard deviation ** Fisher's exact test for qualitative variables, Wilcoxon test for quantitative variables

	Total N=83			(0-2): No substantial bowel damage (n=50)			[>2]: Substantial bowel damage (n=33)			
Duration of symptoms	n	Mean	SD*	n	Mean	SD*	n	Mean	SD*	P**
Total	67	21.6	24.5	43	20.6	24.9	24	23.3	24.2	0.293
<18 months	41	61.2		27	62.8		14	58.3		0.796
> 18 months	26	38.8		16	37.2		10	41.7		

Predictors of Advanced Bowel Damage

**Figure 2:**
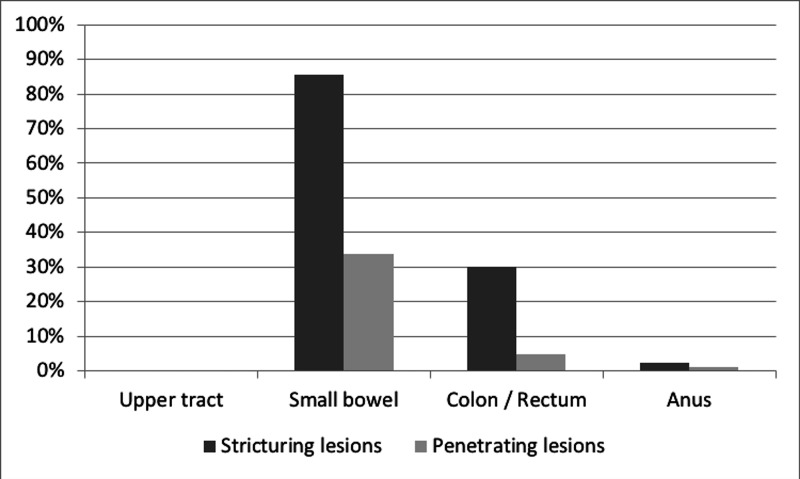
Percentage of damage components for each organ with stricturing or penetrating lesions

After stratifying patients according to the degree of bowel damage, advanced bowel damage showed no difference between females and males (57.6% (n=19) vs. 42.4% (n=14), P=0.30)). A global significant association was found between the penetrating disease phenotype and advanced bowel damage (P=0.01) (Table 4). Two-by-two comparisons revealed that there was a predominance of B1 in the Lémann (0-2) group as compared to the B3 phenotype group (60.0 vs. 16.0%) while in the Lémann >2 groups, the B1 phenotype was proportionally lower than the B3 (30.3% vs. 42.4%) (P=0.003). Stricturing lesions were found more commonly across the small bowel, colon, rectum, and anus than penetrating lesions, as illustrated in Figure 2.

**Table 4:** Comparison of the Lémann score according to the Montreal classification of Crohn’s disease * Fisher's exact test

		Total			(0-2): no substantial bowel damage			[>2]: substantial bowel damage		P*
		N=83			N=50			N=33		
		n	%		n	%		n	%	
Gender										0.302
	Male	41	49.4	27		54.0		14	42.4	
	Female	42	50.6	23		46.0		19	57.6	
Crohn's phenotype (P)										0.011
	B1	40	48.2	30		60.0		10	30.3	
	B2	21	25.3	12		24.0		9	27.3	
	B3	22	26.5	8		16.0		14	42.4	
Crohn’s age category (A)										0.312
	A1	26	31.3	18		36.0		8	24.2	
	A2	52	62.7	28		56.0		24	72.7	
	A3	5	6.0	4		8.0		1	3.0	
Location of disease (L)										0.637
	L1	29	34.9	18		36.0		11	33.3	
	L2	8	9.6	6		12.0		2	6.1	
	L3	46	55.4	26		52.0		20	60.6	

## Discussion

This study assessed the degree of bowel damage at the time of diagnosis of CD in a cohort of Saudi Arabian patients. MRE was used as the imaging modality of choice in conjunction with endoscopic findings to calculate the LI score, assessing for disease severity. Our data suggest that a large proportion of CD patients (39.8%) present with advanced bowel damage at the time of diagnosis, reflecting a severe form of CD. Penetrating disease behavior and ileocolonic involvement were predictive of bowel damage, as shown through the LI scores. To the best of our knowledge, this is the first study to make such an observation.

Previous studies have highlighted the validity of the LI as a tool to identify patients with severe progressive bowel damage and to monitor CD patients to predict the development of complications, as well as evaluate various therapeutic interventions [9-13]. Yet, there are limited data on the use of the LI score as part of the assessment of bowel damage at the time of diagnosis. Liu et al. have described a correlation between the LI score at diagnosis and the risk of surgical intervention within one year of follow-up in patients with CD [10]. They also found that patients with high LI scores at the time of diagnosis had a significantly increased risk of surgical intervention within one year of diagnosis. Gilletta et al. have also studied the importance of evaluating bowel damage at the time of diagnosis in order to predict subsequent substantial bowel damage in CD patients [14]. The authors found that the median LI score was representative of advanced bowel damage at the first evaluation, with further elevations of the LI score showing a strong correlation with intestinal resections. They also confirmed that a cut-off value for the LI score of 3.7 at the time of diagnosis is predictive of an increased surgical intervention risk [10]. A cut-off value of 2 for the LI score was used to determine the presence of advanced bowel damage in a study by Gilletta et al. [14]. In the present study, a mean value of the LI score of 2.4±2.6 was calculated, with 39.8% of patients exhibiting a LI score indicative of advanced bowel damage (of LI score>2) at the time of diagnosis.

Location and behavior of the disease are important factors and have been associated with the severity and complexity of CD [15-17]. In the present study, 60.6% of patients who exhibited an LI score that is suggestive of advanced bowel damage presented with ileocolonic involvement at the time of diagnosis, and 42.4% had a penetrating disease phenotype. Advanced bowel damage was not associated with a younger age at the time of diagnosis. This is contrary to what was proposed by Lunder et al., where a significant association was found between LI scores at the time of diagnosis and a complicated disease behavior in younger patients [18].

CD is traditionally believed to cause bowel damage over time. Hence, the duration of the disease is often considered to be a significant factor in its association with the progression of bowel damage [19-20]. Our study found that a shorter disease duration, of less than 18 months, shown in 62.8% of patients, was not associated with the absence of bowel damage. This is contradictory to the findings reported by Rispo et al., which assessed the duration of disease as a predictor of LI scores [11]. They noted that shorter disease duration was significantly associated with the absence of bowel damage when compared to patients with substantial bowel damage that had longer disease duration. In the present study, the absence of a correlation between the duration of symptoms and the degree of bowel damage could be an indicator that Saudi patients with CD present with a severe phenotypic form that is different from those observed in some or all other parts of the world and highlights the importance of baseline risk stratification [20].

We described for the first time, to the best of our knowledge, the degree of bowel damage assessed by LI scores at the time of diagnosis for CD patients among Saudi Arabian CD patients. We believe that this will facilitate a greater understanding of LI as the most reliable current modality for the assessment of CD and for predicting the severity of cumulative bowel damage. We are hopeful that this can help in planning different treatment strategies at an early phase of the disease. Our study is limited by its retrospective nature, which relies on existing patients’ medical records that often had missing values, which may have diminished the accuracy of the results. Consequently, further limitations, such as incomplete documentation, loss of follow-up reports, and lack of a unified patient’s medical profile in hospitals in Saudi Arabia are intrinsic constraints.

## Conclusions

This Saudi Arabian cohort of patients with CD often presented with advanced bowel damage at the time of diagnosis, suggesting a possible severe form of CD in this group of patients that may be endemic to Saudi Arabia. Frequent evaluations and assessments over time are thus needed to help better understand the nature of the disease and to make better-informed decisions on treatment policies and patient care.
